# The Neonatal Comfort Care Program: Origin and Growth Over 10 Years

**DOI:** 10.3389/fped.2020.588432

**Published:** 2020-10-30

**Authors:** Charlotte Wool, Elvira Parravicini

**Affiliations:** ^1^York College of Pennsylvania, College of Nursing and Health Professions, York, PA, United States; ^2^Division of Neonatology, Department of Pediatrics, Columbia University Irving Medical Center, New York, NY, United States

**Keywords:** interdisciplinary care, program development, life limiting conditions, perinatal palliative care (PPC), neonatal palliative care

## Abstract

The objective of perinatal palliative care is to provide holistic and comprehensive health care services to women who are anticipating the birth of a neonate diagnosed prenatally with a life-limiting condition and to continue supportive interventions for the mother and neonate after the birth. The nature of pregnancy, with two patients requiring medical care, requires clinicians from different specialties to engage with one another, the patient, and her chosen family members. Following birth, additional skill sets to treat the medical and comfort needs of the neonate, as well as the psychoemotional and medical needs of the mother, are required. An interdisciplinary team is necessary to assist families throughout the pregnancy and postnatal journey, and coordination of such care is an integral component of palliative care services. The number of palliative care programs is increasing, but little is written about the origins of such programs, their subsequent growth, and how transitions of care occur within the programs. In this publication, we will present data garnered from interdisciplinary team members of a single organization, the Neonatal Comfort Care Program at Columbia University Irving Medical Center, and how they provide care for families throughout the pregnancy and postnatal trajectory. We will address the origin and growth of the program, the development of the interdisciplinary team, and the strategies used for high-quality communication and their respective impact on care continuity. We will also provide specific recommendations from data gathered from team members, examine the role of formal and informal education, and identify barriers and future opportunities.

## Introduction

There is rarely a time in a family's life that entails more transitions than pregnancy and the birth of a child. It is a time of anticipation, changes, preparation, and often great joy. When a woman is faced with news that her expected child has a life-limiting condition (LLC), the pregnancy course abruptly changes, as do the complexity and challenges she and her family experience. Historically, women were offered the options to terminate the pregnancy or prepare to have their neonate admitted to an intensive care unit. However, in 1997, the concept of perinatal hospice was introduced (1), followed by clinical recommendations for a neonatal end-of-life palliative care protocol (2). Since then, perinatal palliative care (PPC) programs, which are defined as comprehensive, interdisciplinary, coordinated services offered from fetal diagnosis through the neonatal period, have experienced significant growth (3) and national recognition (4–6). This extension of a woman's right to choose respects patients' cultural beliefs and values, can empower women, and has been met with increases in patient satisfaction (7, 8).

Clinicians from many disciplines play an integral part in supporting families by providing compassionate, evidence-based care and can offer information and services that allow families to make the most of the time with their neonate. Data show that women who opt to continue a pregnancy with an LLC appreciate opportunities to emotionally adjust and report having no regrets about their decision (9, 10). A variety of interventions, including anticipatory guidance, prenatal consults, early and continuous bereavement support, and coordination of care allow families to begin the difficult but important process of adjusting to the implications of an LLC (11, 12). Formal, integrated palliative care programs have structures in place that ensure the interdisciplinary team (IDT) can communicate well, manage the needs of families, and offer high-quality care.

The purpose of this article is to provide practical information for professionals interested in starting, sustaining, or increasing PPC services within an organization by using the model of the Neonatal Comfort Care Program (NCCP) at Columbia University Irving Medical Center (CUIMC). Aims are to (1) provide an overview of the program's origin and its subsequent growth, (2) explain the formation of the IDT, (3) outline communication strategies of team members to ensure continuity of care, (4) highlight recommendations from team members working in the program, (5) emphasize the significant role of education in culture change, and (6) identify barriers in developing the program and future opportunities.

## Aim 1—Program Origin and Subsequent Growth

**Figure 1:**
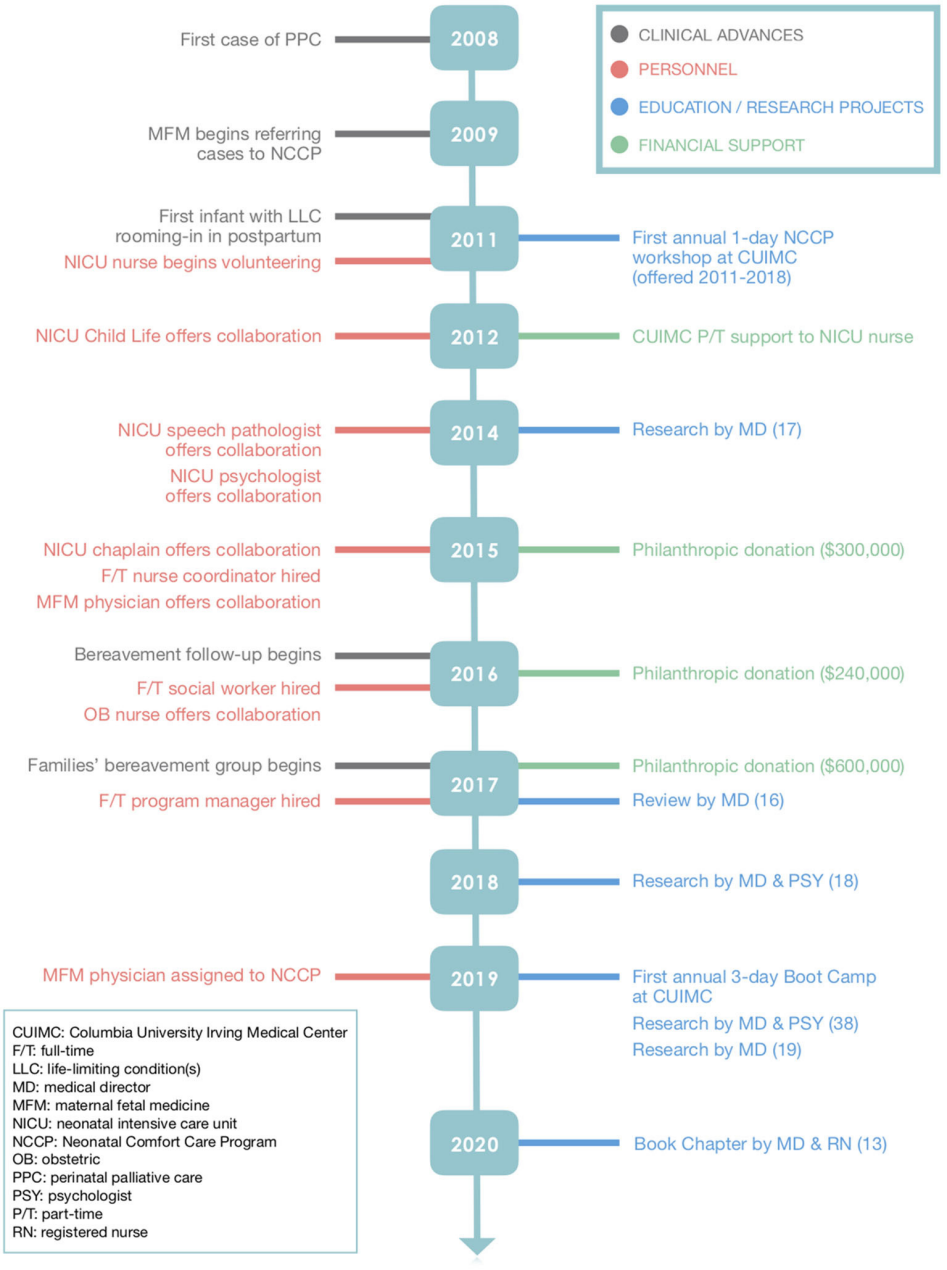
Timeline of Neonatal Comfort Care Program (NCCP) Growth and Milestones.

CUIMC is a large academic institution with ~4,000 deliveries annually and about 800 pregnancies involving fetal anomalies, including LLC. The NCCP began in 2008 and grew out of a desire to provide the most comfortable and loving environment for neonates diagnosed *in utero* with LLC, help families manage and navigate the immense practical and emotional burdens associated with LLC, and offer an option of care that families identify as safe, supportive, and in sync with their personal beliefs and desires. The idea for formalizing a strategy to provide comfort care for neonates with LLC started with a neonatologist. At the beginning, obstetric and maternal fetal medicine colleagues were invited to make referrals for a prenatal palliative care consult whenever a candidate was identified. As families provided positive feedback of their experiences and as relationships between neonatology and obstetric providers advanced, referrals increased. Consults are medically oriented meetings wherein the physician reviews the patient's medical history; meets with family members to assess their understanding, desires, and values; and makes recommendations about care and treatment. The consults are reimbursable. Patient encounters are supportive services provided by appropriate IDT members and are tailored to family needs. Figure 1 provides a timeline outlining the onboarding of the NCCP members, educational programs, acquisition of funding, and contributions to the literature.

Annual prenatal consults have grown logarithmically from 13 consults in 2008 to 64 in 2019. Patient encounters have increased in a similar fashion from 16 in 2008 to 127 in 2019.

## Aim 2—Development of the Core and Interdisciplinary Teams

In 2011, a neonatal intensive care unit (NICU) nurse self-identified an interest in partnering with the neonatologist, leading to a part-time position in 2012. By 2017, as professionals expressed an interest, a core team was established with a medical director from neonatology dedicated to, but not funded by, the NCCP program. A full-time nurse, social worker, and program manager were funded from unsolicited philanthropic donations given by individuals and foundations. The program manager is focused on program development and outreach to establish sustainability. The IDT includes collaborators/champions from neonatology and obstetrics, a child life specialist, speech language pathologist, psychologist, chaplain, obstetric nurse, and maternal–fetal medicine physician. The uniqueness of the program lies in an intentional continuity of care among team members and professionals and a desire to ensure quality services throughout the entire perinatal journey.

During this decade of growth, the NCCP established and published innovative clinical guidelines focusing on the comfort of the neonate and family bonding, leading to an impressive culture change (13). One of the key elements of bonding is that the mother and her infant should have close contact and not be separated. In 2008, in our institution, as elsewhere, neonates with LLC were admitted to the NICU for “comfort care,” resulting in separation of the mother and infant. Intentional in-house education was offered, and by 2018–2020, all families followed prenatally by the NCCP received the option to room-in after birth, and all mothers who elected this option (98%) successfully kept their infant with them in a private postpartum room. The NCCP offers rooming-in for infants at the end of life, as well as infants with any kind of LLC, as long as the parent(s) desire palliative care management. Oxygen via nasal cannula, nasogastric or syringe feeding, and pain management are provided, and the NICU team is responsible for the infant. Another essential element for bonding and comfort is feeding; involvement of a speech pathologist has been key to safely and successfully feeding these fragile neonates throughout their natural lives.

## Aim 3—Communication Strategies

### Referrals for Consultation

Referrals for consultation to the NCCP are initiated through (1) obstetricians directly initiating a consult with the NCCP; (2) identification of new patients in weekly meetings among the NCCP medical director, nurse coordinator, and maternal–fetal medicine and neonatology physicians; and (3) self-referral of women requesting a PPC consultation in partnership with their obstetric provider.

### Candidates

Candidates include pregnant women with potential or certain fetal LLCs and those with a complex fetal diagnosis with a potential adverse prognosis. A specific list of fetal LLC diagnoses triggers a referral to the NCCP. Included are conditions with expected early demise, such as renal agenesis, anencephaly, and limb-body-wall syndrome, as well as cases in which the burden of intensive care may exceed benefits, such as trisomy 13, trisomy 18, genetic syndromes with life-limiting prognoses, extreme prematurity at the cusp of viability, or complex congenital heart disease. Complex conditions with potential adverse outcomes also initiate a consult. Examples include uncertain diagnoses, such as multiple skeletal anomalies or severe brain anomalies, and known diagnoses with uncertain prognosis, such as bilateral renal dysplasia, severe lower urinary tract obstruction, or severe hydrops. The NCCP takes medical care of all infants with these conditions. Regardless of the family's decision to have comfort care vs. a trial of intensive care vs. full NICU support, the NCCP is available to support the family throughout the perinatal journey. At birth, if the family elects comfort care, the NCCP takes direct care of the infant.

### Consults and Patient Encounters

Once a consult is initiated, the medical director is notified, and the first meeting with the family is planned. The medical director, nurse coordinator, and social worker meet with the family during the first consult to (1) probe the family's prognostic awareness, (2) clarify diagnosis, prognosis, (3) offer options for postnatal plans, (4) propose supportive interventions, and (5) establish a relationship of trust that will continue along the entire perinatal journey. Two aspects of care planning occur in tandem. First, the medical director oversees a postnatal medical care plan dependent on fetal gestational age and family need. Second, the family will meet with the nurse coordinator and the social worker, who offer supportive interventions and resources during pregnancy, delivery, and beyond. The patient encounters consist of interventions such as anticipatory guidance, discussion and documentation of a family birth plan, psychological counseling, memory making, sibling support, religious and cultural rituals, and referrals to other IDT professionals as needed. Usually two to four meetings occur.

### Communication

A systematic approach of communicating patient events occurs through a weekly meeting among the core team and the maternal–fetal medicine physician. The outcome of these clinical rounds is reported in a weekly “comfort care list” emailed to the IDT and NICU and obstetric fellows. In addition, documentation from each consultation is reported in the electronic health record by each member of the NCCP team. The core team also meets monthly to review follow-up of neonates discharged home or to hospice and the follow-up of mothers/families who suffered a perinatal loss. In addition to these formal communication processes, informal conversations among team members occur through telephone calls, texting, and face-to-face meetings. The team is careful to apply patient privacy standards when communicating.

### Continuity of Care—Prenatal to Postnatal

One outcome of high-quality communication among team members is the opportunity to provide continuity of care. In the NCCP, the medical director and the nurse coordinator, when available, are present at delivery to ensure continuity of care to the family or to mentor staff members in the obstetric and neonatal units. The champion obstetric nurse is also available to mentor and facilitate care in the delivery room and postpartum unit. The unpredictability of labor requires a communication system that allows pristine fulfillment of the medical and interdisciplinary birth plans.

The number of newborns with LLC is relatively small so the medical director assumes direct care of most neonates until death or discharge, along with the NICU staff (NICU fellow, nurse practitioner, resident). When the neonate is admitted to postpartum, a NICU nurse is assigned to assess the infant once a shift or more often if needed. IDT members are consulted as needed. A child life specialist helps with memory making and sibling support, and the chaplain addresses spiritual needs and coordinates specific parent-requested rituals. Standardized guidelines focused on achieving a state of comfort for the neonate are implemented and have been published (13).

The NCCP social worker collaborates with the obstetric social worker in supporting the mother and the family. The social workers provide resources and information about a coordinated discharge to home, follow-up with hospice, and disposition of the infant. Once the mother is discharged home, the care coordinator and social worker, and other IDT members if needed, continue to support the family by offering a bereavement follow-up for at least a year.

## Aim 4—Recommendations From Team Members

Data regarding recommendations for those interested in starting or developing a program were collected from individual team members in the NCCP program. Recommendations were collated and are delineated in Table 1.

**Table 1:** Recommendations and suggested resources from NCCP team members to clinicians planning to start a PPC program.

**CORE TEAM**	
Neonatologist, program director	• Create a mission and vision and access it to maintain motivation (14) • Establish collaboration with OB • Look for “champion” professionals interested in collaboration • Work cohesively and regularly with team members to ensure optimal communication • Use professional encounters to teach and role model the tenets of PPC (15, 16) • Establish policy/guidelines for PPC in your institution (12) • Measure and report program outcomes and successes (17–19)
Registered nurse, program coordinator (former NICU RN)	• Identify champion RNs interested in PPC and provide teaching and mentoring • Participate in online and live educational opportunities (15) • Use online and community resources to gather bereavement supplies (20) • Develop a curriculum of Nursing PPC to create a PPC mindset
Program social worker (former PICU SW)	• Document all tasks related to PPC and build a case for SW position with data • Join perinatal hospice (20) network and network with other SWs to gain insights and information (21) • Participate in online and live educational opportunities (15, 22)
**IDT TEAM**	
OB registered nurse	• Develop QI projects to build a case for resources for PPC • Train and mentor OB nurses to create a PPC mindset (23, 24) • Network with OB nurses in other institutions to share ideas and resources for PPC
MFM physician	• Collaborate with the team in pregnancy management to achieve family's expectations (4) • Train OB staff to respect and support the family's goals (25, 26) • Develop research projects to demonstrate the benefits of PPC
NICU speech-language pathologist	• Contact experienced colleagues for advice (27) • Consider training champion NICU RNs to assess feeding needs for a neonate and become familiar with specific feeding equipment • Train all staff to respect and support the family's goals relative to providing nutritive or non-nutritive therapies (28)
NICU child life specialist	• Train other child life specialists to specific interventions in PPC (29) • Consider training “champion” RNs to become familiar with memory making during pregnancy and at birth
NICU psychologist	• Contact in-house psychologists and assess interest in working with bereaved parents • Develop collaboration with team SW • Be intentional about listening to parents and help them navigate their experiences during pregnancy and beyond (30, 31)
NICU chaplain	• Partner with in-house or community faith leaders • Familiarize clinicians with spiritual screening using FICA Spiritual History Tool (32) • Assess patient's spiritual preferences prenatally to prepare for spiritual interventions (33, 34)

## Aim 5—Education to Produce Culture Change

Education has been pivotal to the advancement of PPC in CUIMC. Both informal and formal educational strategies have promoted a culture change and facilitated a positive PPC mindset among CUIMC personnel, as well as clinicians in the surrounding metropolitan areas. Neonatal palliative care advancements set the stage for the uptake of PPC with the downstreaming of literature and the increased interest among neonatologists to assist and prepare parents expecting a neonate with an LLC.

Starting in 2014, the NCCP organized and offered an annual 1-day workshop drawing 50 to 70 professionals from CUIMC and the surrounding region each year. The workshop included lectures, discussion of clinical cases, and parent interviews. In 2019, the workshop was expanded to a 3-day boot-camp training course with continuing education credits for physicians and nurses. The program agenda is available on the website and included formal lectures, role-modeling case studies, hands-on demonstrations, group discussion, parent voice, and networking opportunities. More than 80 participants from 19 states and several countries attended. Unique to the program was the opportunity for each NCCP team member to provide evidence-based strategies and share their clinical experiences with the participants (15).

Informal education occurs routinely by the nurse coordinator and champions within the IDT. Examples include bedside teaching in the delivery room, postpartum unit, and the NICU as mothers and their neonates present to these areas. The obstetric nurse champion conducts huddles, mentoring, and bedside teaching and is instrumental in keeping lines of communication clear and consistent. Information about the NCCP is given to all new nurses during orientation.

The medical director of the NCCP is responsible for training and mentoring obstetric and neonatal fellows and residents in the clinical setting. Additionally, a five-lesson curriculum was developed and is used to formally present essential elements of PPC.

## Aim 6—Barriers and Future Opportunities

Barriers to implementing workplace change were two-fold. First, PPC is an emerging field, and the evidence base was limited at the start of the NCCP program. Coupled with the development of new research knowledge were positive clinical experiences. Over time, resistance to PPC diminished, the culture improved, and the mindset of perinatologists changed. A second barrier was limited funding. Resources were allocated over time and provided support for new staff, educational initiatives, and research endeavors.

The future of the NCCP program is promising. Educational goals of the NCCP will build upon the foundation created in the last decade. Plans are in place to increase educational outreach and establish deeper collaborations with regional institutions to assist with replicating best practices related to PPC. Educational recommendations from NCCP team members include additional in-services, development of a basic curriculum for nurses centered on area of expertise, and establishment of a PPC fellowship for NICU, obstetrics, and social work. Expansion of the clinical team includes plans for a dedicated midwife who would provide continuity of care during the perinatal journey, facilitate communication during encounters with various obstetrics providers, offer one-on-one childbirth education, be present at the birth, and provide postpartum follow-up and lactation support. Involvement of additional clinicians to act as NCCP champions will reinforce expertise, assist with training and supporting colleagues, and provide more penetration of PPC culture within units. Ongoing mental health treatment in the form of groups and/or individual/couples therapy is also under consideration. Further research will provide new knowledge upon which evidence-based practices can be established.

## Discussion

PPC literature has grown in the last two decades and, with it, application into clinical practice. Palliative care programs are in 25 countries worldwide, with 246 in the United States and 67 globally (20). However, of the 246 perinatal hospices in the United States, only 20% offer hospital-based perinatal care, and nearly 40% offer only bereavement support. Of the 20% offering hospital care, the NCCP is unique in its prioritization of uninterrupted support of families of neonates with LLC. From the moment of diagnosis, through birth and beyond, the same physician and team members provide continuity of care to the neonate and family throughout each step of the journey.

As palliative services expand to the perinatal population, patient- and family-centered care will continue to optimize quality of life by anticipating, preventing, and treating suffering (35) through the continuum of the pregnancy and postnatal course. There are several benefits of a formal program as team members optimize communication strategies, work within standardized guidelines, and track outcomes and quality care. Systematic incorporation of evidence into practice settings can take many years, and over time, with dedicated staff and administrative support, referrals increase. The work of improving quality and effectiveness of health services requires consistent vision of individuals and teams, along with resources and administrative support, to solidify lasting change.

Culture change is an essential part of clinical advancement, especially with respect to palliative care as palliative therapies are interventions with complexities that affect the physical, psychoemotional, and spiritual aspects of human care. Advances in the NCCP program, including establishment of guidelines available to any professionals involved in perinatology, occurred through multifaceted approaches, including a mindset reorientation for leaders and frontline clinicians. As more clinicians become aware of and are supported in providing palliative care, their confidence increases, and a significant cultural shift occurs.

In line with current recommendations, an IDT should consist of a multitude of experts, all of whom play specific roles in providing tailored services to neonates, women, and their family members. The essential first step is the collaboration between the obstetric and neonatology services, which ensures that pregnancy and delivery are managed in a continuum (4). Anecdotally, medical directors are commonly neonatologists or obstetricians rather than palliative care physicians. These specialists are housed in every hospital in the USA whereas palliative care physicians are not. Essential elements of PPC come under the auspices of obstetrics and neonatology who have expertise with prenatal counseling, pregnancy management, and palliative comfort interventions for infants with a shortened life span. Communication is optimized through a systematic, consistent collaboration within the IDT. Unlike several programs in the US (3) led by a nurse coordinator or a bereavement counselor, the NCCP was initiated and developed by a neonatologist who established a collaboration with obstetricians and provides prenatal counseling and direct medical care to the neonates, promoting continuity of care for each family. The nurse leader facilitates the nursing aspects of care and coordinates interdisciplinary services, helping to form a cohesive approach to care. Thanks to professionals offering their services and prioritizing rooming-in with mother and feeding as comforting to the neonate, the NCCP has been building a mindset that contributes to the creation of a peaceful, safe, and loving environment. The life of the neonate, rather than the anticipation of his or her loss, becomes the focus of attention.

Data provided by NCCP team members offer recommendations to read appropriate discipline-focused literature, attend educational events, and listen to parents. The identification of local “champions” is key to establish the much-needed culture change toward a mindset open to PPC among professional working in the perinatal field. Professional networking affords a host of benefits by strengthening and expanding the exchange ideas and information. Networking can also facilitate change through brainstorming and problem solving. Lastly, PPC is still a very young field in medicine and practice needs to be evidence-based through prospective research.

Although the number of PPC programs has been increasing over the past years, training and education for professionals have not kept pace. Evidence-based, consistent, comprehensive training courses for professionals involved in PPC across disciplines are not widely available. A lack of knowledge is linked to clinician discomfort in providing care for families affected by an LLC (36). Since the start of NCCP, educational initiatives were a priority, leading to the changing dynamics among units that forged cultural and clinical changes. Educational approaches were diverse and included formal annual training events, group lectures, and bedside training and mentoring.

The allocation of resources is a constant challenge for health care administrators, and yet, the lack of resources can have serious consequences on patient-centered quality care (37). A variety of approaches has helped keep the NCCP operational, including the strong motivation of the core team members to provide comfort care despite scarce resources, the availability of some IDT members to offer some extra hours, and a cohesive team mentality enabling optimal communication and shared successes. Plans to continue outreach to philanthropic foundations to ensure sustainability are a future priority.

## Conclusion

Building a new program and changing organizational culture take strong motivation, commitments of time and energy, and, eventually, adequate funding. Clinical leaders can forward a consistent vision and focus on creating an ongoing narrative related to optimal comfort care for neonates with LLC and support for their families.

## Data Availability Statement

The raw data supporting the conclusions of this article will be made available by the authors, without undue reservation.

## Author Contributions

CW and EP conceptualized and designed the report, drafted the initial manuscript, and approved the final manuscript as submitted. Both authors contributed to the article and approved the submitted version.

## Conflict of Interest

The authors declare that the research was conducted in the absence of any commercial or financial relationships that could be construed as a potential conflict of interest.
